# Outcomes of Focused Double Extrastimuli Mapping and Ablation in Ventricular Tachycardia

**DOI:** 10.1111/jce.70196

**Published:** 2025-11-26

**Authors:** Surachat Jaroonpipatkul, Andrea N. Keithler, Thipsukhon Sathapanasiri, Harikrishna Tandri, T. Jared Bunch, Ravi Ranjan, Klitos Konstantinidis

**Affiliations:** ^1^ Division of Cardiovascular Medicine University of Utah Health Salt Lake City Utah USA; ^2^ Nora Eccles Harrison Cardiovascular Research Training Institute University of Utah Salt Lake City Utah USA; ^3^ Department of Pharmacotherapy, College of Pharmacy University of Utah Salt Lake City Utah USA; ^4^ Division of Cardiovascular Medicine Vanderbilt University Medical Center Nashville Tennessee USA

## Abstract

**Background:**

Conventional substrate mapping for scar‐related ventricular tachycardia (VT) often fails to fully delineate critical arrhythmogenic components. Functional mapping strategies using extrastimuli have shown promise but are limited by procedural complexity.

**Methods:**

In total, 57 patients undergoing VT ablation between March 2023 and October 2024 were included in the study. Patients were grouped based on whether targeted double extrastimuli mapping (focused DEMAP) was used to guide mapping and ablation. Mapping was performed using EnSite X with omnipolar technology. Clinical and procedural outcomes were compared between focused DEMAP and non‐DEMAP groups with outcomes follow‐up at 12 months.

**Results:**

Focused DEMAP revealed a significantly higher correlation between DZs and VT critical isthmuses compared to sinus rhythm mapping (75.9% vs. 37.1%, *p* = 0.01). VT‐free survival at 12 months was higher in the DEMAP group (88.03%) than in the non‐DEMAP group (58.10%, *p* = 0.04). Trends also favored DEMAP in reducing cardiovascular hospitalization (10.55% vs. 24.70%) and mortality, though not statistically significant. Overall procedure time was similar mainly driven by less time during ablation and a lower mean ablated area in the focused DEMAP group compared to the non‐DEMAP group.

**Conclusion:**

The focused DEMAP, a focused double extrastimulus mapping strategy, enhances VT substrate identification and is associated with improved VT‐free survival without prolonging procedure time. This approach may refine substrate‐based ablation and optimize lesion targeting without increasing procedural burden.

## 1 Introduction

Identification of ventricular tachycardia (VT) circuit components during sinus rhythm remains a challenge. Substrate mapping focuses on identifying surrogates of the VT isthmuses during sinus rhythm. Various substrate mapping techniques are used in clinical practice, including the identification of low‐voltage areas, late potentials (LPs) [1], local abnormal ventricular activities (LAVAs) [2], and deceleration zones (DZs) using isochronal late activation mapping (ILAM) [3]. However, these methods encounter difficulties in accurately evaluating arrhythmogenic substrates. Importantly, abnormal near‐field electrograms can be obscured by normal far‐field signals during sinus rhythm or right ventricular (RV) pacing, and bipolar voltage readings may vary significantly with pacing from different sites. Slow corridor activity may occur during main ventricular activation at baseline, leading to potential omission of the critical isthmuses from substrate maps using traditional techniques. Signal quality is technology‐dependent, and its interpretation is inherently subjective.

**Figure 1:**
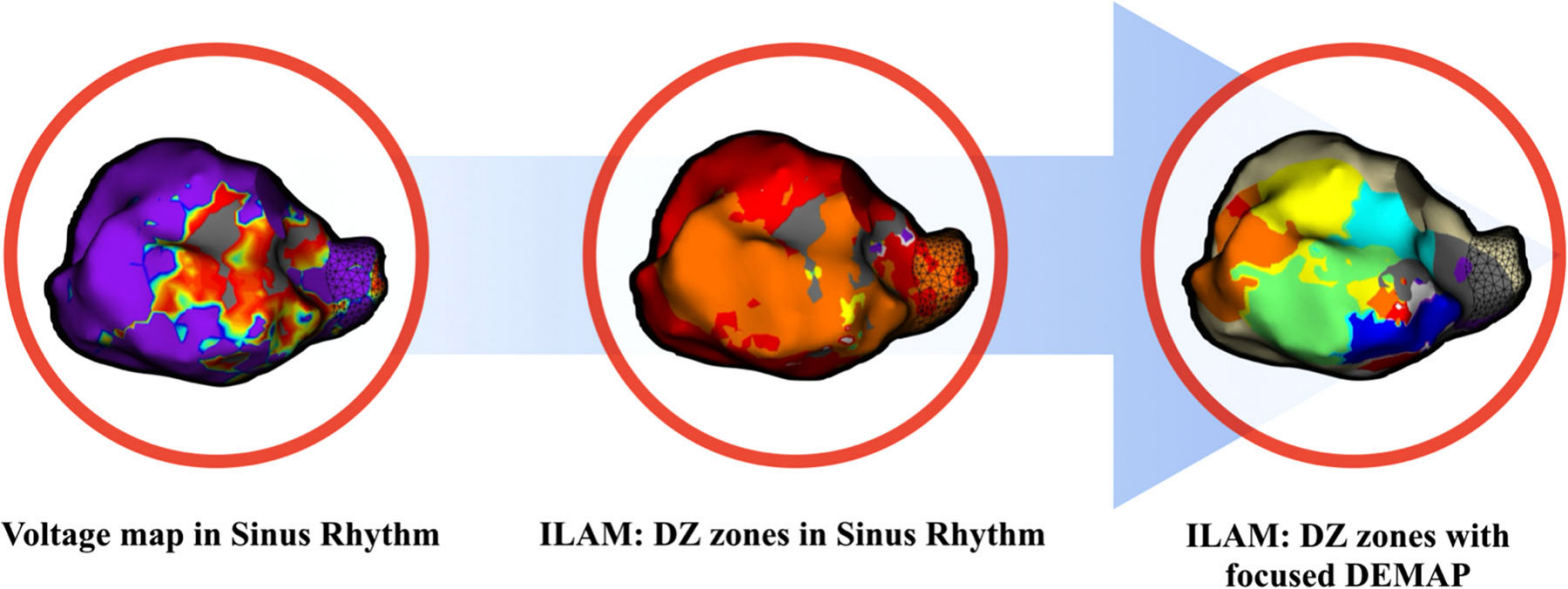
Central illustration of the focused DEMAP protocol: stepwise substrate mapping and functional refinement. This figure outlines the sequential workflow of the focused DEMAP for ventricular tachycardia substrate characterization. The left panel displays bipolar voltage mapping in sinus rhythm to delineate low‐voltage areas corresponding to scar and border zones. The middle panel shows isochronal late activation mapping (ILAM) in sinus rhythm, highlighting deceleration zones suggestive of conduction slowing. The right panel demonstrates refinement with targeted ILAM following double extrastimuli pacing (focused DEMAP), revealing functional conduction channels not apparent during baseline mapping. This approach facilitates more precise identification of critical isthmuses for ablation.

To address these limitations, functional substrate mapping techniques utilizing one or more extrastimuli during baseline rhythm have been developed, potentially offering greater specificity for detecting VT isthmuses [4, 5]. Studies employing whole‐chamber double extrastimulus protocols have demonstrated promise in VT substrate identification. A recent study of whole‐chamber S3 mapping showed improved identification with S3 compared with S2 or S1 alone, including a greater number of DZs, and suggested that this method may improve VT ablation success [6]. Whole‐chamber mapping can increase procedure duration, increase exposure to prolonged extrastimuli testing that may be proarrhythmic, and may offer limited benefit compared to a more focused anatomic approach guided by established VT substrate localization methods. Building on this concept, we present our findings using a targeted double extrastimulus mapping (DEMAP), which we refer to as the focused DEMAP. In this protocol, we used focused DEMAP in areas of interest to unmask, identify, and eliminate critical areas of the VT circuits (Figure 1). This targeted method is designed to refine substrate identification, improve specificity in detecting VT isthmuses, guide ablation strategies, and enhance ablation outcomes without requiring extensive scar homogenization.

We hypothesize that an ablation strategy guided by the focused DEMAP can achieve improved procedural success and VT recurrence‐free survival by selectively identifying critical areas of arrhythmogenic substrates compared with conventional substrate mapping techniques.

## 2 Methods

### 2.1 Patient Selection and Study Design

This nonrandomized study included consecutive patients referred for catheter ablation of scar‐related sustained VT at the University of Utah Hospital between March 2023 and October 2024. Patients in the early part of the study period underwent sinus rhythm mapping alone who acted as the control group. These patients were compared with subsequent patients who underwent focused DEMAP in addition to sinus rhythm mapping.

Eligible participants were adults aged 18 years or older with structural heart disease, including ischemic cardiomyopathy (ICM) or nonischemic cardiomyopathy (NICM). The diagnosis of ICM was based on a history of myocardial infarction with Q waves, focal wall motion abnormalities observed on imaging, or fixed perfusion defects correlating with coronary stenosis or prior coronary intervention. NICM was characterized by patient history and the presence of myocardial scarring that did not follow a coronary vascular distribution and without evidence of coronary artery disease on imaging. Patients with idiopathic VT, including outflow tract VT, fascicular VT, and ventricular fibrillation (VF), as well as those who underwent epicardial access, were excluded from the study.

During the study period, double extrastimulus mapping focused on the region of interest was introduced as a functional substrate mapping technique and began to be used for patients referred for VT ablation as a complement to the standard mapping approach. Patients were retrospectively categorized into two groups: focused DEMAP and non‐DEMAP, based on the mapping approach used to guide identification of the critical isthmus and ablation. The non‐DEMAP group included patients who underwent traditional substrate and/or VT activation mapping and ablation. In the focused DEMAP group, in addition to traditional mapping performed in the non‐DEMAP group, a focused S3 extrastimuli was performed in the regions of interest guided the ablation strategy and used as an endpoint elimination of the slow conduction zones identified during focused S3 mapping in addition to non‐inducibility. Regions of interest were primarily defined as areas of dense scar and border zone. In addition, regions outside these predefined areas that demonstrated abnormal electrogram characteristics (e.g., fractionation, local delay, or multicomponent) were also assessed using focused DEMAP.

Demographic, procedural, and follow‐up data from both groups were collected for analysis and comparison of the two mapping approaches. We then evaluated the efficacy and clinical outcomes of VT ablation using targeted double extrastimuli mapping compared to VT ablation procedures using traditional substrate and/or VT activation mapping techniques over a follow‐up period of up to 12 months.

The study was conducted in accordance with the Declaration of Helsinki, with ethical approval obtained from the Institutional Review Board of the University of Utah Medical Center. Written informed consent was obtained from all participants before enrollment. Data supporting the study's findings are available from the corresponding author upon reasonable request.

### 2.2 Procedure Details

Patients were brought to the cardiac electrophysiology laboratory in a fasting state and received sedation with monitored anesthesia care with standard monitoring as well as serial arterial blood gases (ABGs) and lactate levels throughout the duration of the procedure. All procedures were performed by a single operator (K.K.). Endocardial access to the left ventricle (LV) was achieved via either a transseptal or retrograde aortic approach, at the operator's discretion. All patients underwent traditional substrate mapping, which was performed using the Advisor HD Grid Mapping Catheter Sensor Enabled (Abbott, Abbott Park, IL) with the EnSite X mapping system, incorporating omnipolar technology (OT) for enhanced mapping resolution. Mapping was completed during sinus rhythm or with RV pacing in cases where intrinsic atrioventricular conduction was absent.

### 2.3 Standard Substrate Mapping

High‐density whole‐chamber voltage mapping was performed during sinus or RV paced rhythm in conjunction with ILAM to delineate low‐voltage regions and identify DZ based upon the presence of isochronal crowding [7, 8]. In this study, bipolar and omnipolar substrate maps were annotated using a voltage range of 0.1–1.0 mV. For unipolar mapping, we applied chamber‐specific thresholds: 1.0–5.5 mV for the right ventricle (RV) and 1.0–8.3 mV for the LV. The boundaries of DZs were drawn 5 mm beyond the isochronal crowding zone, and their total surface area was measured to quantify the extent of conduction slowing within the mapped region. Candidate DZs were evaluated through meticulous manual assessment of electrograms to confirm discontinuous fractionated characteristics or split local activation. Real‐time verification by the operator ensured consistency with local activation timing at neighboring sites within a 1 cm radius. Electrograms that lacked reproducibility or corroboration from adjacent signals were excluded from the analysis. If mapping density was insufficient or abnormal electrograms were indistinguishable from noise, the operator remapped the region to ensure comprehensive evaluation. Automated annotation algorithms were used to detect LPs and LAVAs, which were manually reviewed and adjusted to ensure accuracy. Their total surface area was calculated by summing the areas of all identified zones. These annotations were further validated post‐procedurally to refine substrate identification. Limited pace mapping was performed to confirm findings within DZs. In this study, all DZs were identified using omnipolar electrograms obtained with the Advisor HD Grid on the EnSite X system, and all figures in this manuscript represent omnipolar data.

For patients with inducible VT, the critical isthmus was identified on the electroanatomic map by evaluating the presence of diastolic potentials, entrainment with a post‐pacing interval minus the tachycardia cycle length of < 30 ms with concealed fusion, termination of VT during ablation, or the most suitable pace map location showing the longest stimulus‐to‐QRS interval or closest match to VT morphology in cases where entrainment was not feasible. These criteria helped differentiate the critical isthmus from other mapped regions, providing specific targets for ablation.

### 2.4 The Focused DEMAP

**Figure 2:**
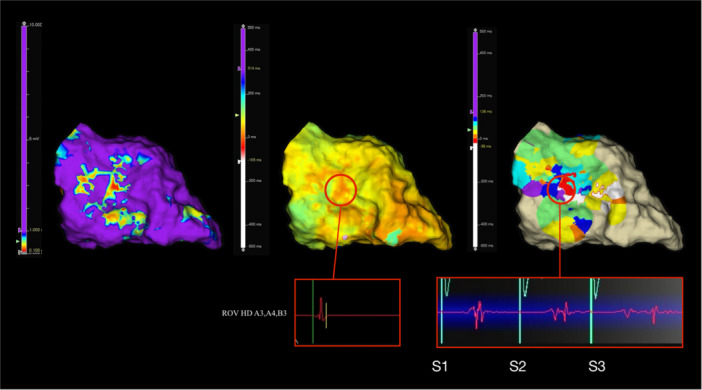
Electroanatomic mapping progression using the focused DEMAP protocol. This figure illustrates the mapping process in a patient undergoing VT ablation with the focused DEMAP protocol, demonstrating the transition from baseline voltage mapping to functional substrate assessment with double extrastimuli. All electrograms and maps are obtained with omnipolar mapping. (left) Baseline voltage mapping during sinus rhythm, with voltage thresholds set at 0.1–1.0 mV. (middle) Isochronal late activation mapping (ILAM) during sinus rhythm, displaying activation timing across the mapped region. The area marked with a red circle initially shows relatively discrete electrograms with minimal fractionation or late potentials. (right) DEMAP mapping with double extrastimuli, revealing additional functional abnormalities. The same region marked in the middle panel now exhibits fractionated electrograms and late potentials, suggesting unmasking of slow conduction that was not apparent in sinus rhythm.

The ventricular effective refractory period (VERP) was determined via pacing from the RV apex. Once the ERP was established, the S2 was increased by 50 ms, and an additional extrastimulus (S3) was introduced up to 30 ms above the ERP. We then performed an evaluation of the functional substrate with targeted S3 mapping in areas of interest following standard substrate mapping. Extrastimuli (S2–S3) were delivered from the RV apex, and activation was mapped within targeted regions of interest during this pacing. The window of interest (WOI) was manually adjusted in each case to capture delayed or fractionated electrograms after S3 pacing. The EnSite X system allows detection of signals up to 500 ms to either side of the reference “zero” line, enabling inclusion of significantly delayed potentials while avoiding annotation of signals from subsequent beats outside the pacing protocol. In all patients of the DEMAP group, standard ILAM in sinus rhythm was also performed as part of the substrate mapping protocol. The area of interest generally included the dense scar and the scar border zone. In the area of interest, we performed focused DEMAP to enhance the identification of arrhythmogenic substrates by unmasking regions of slow conduction and potential corridors of the VT circuits (Figure 2). Local activation times were automatically determined based on the latest deflection of electrograms, utilizing the system's last deflection algorithm. Both bipolar and omnipolar voltage maps were acquired for validation and comparison; however, only omnipolar maps were used for defining DZs and guiding ablation, given their orientation‐independent voltage measurements and higher spatial resolution.

### 2.5 Ablation Strategy

Ablation was performed using an irrigated tip catheter (FlexAbility SE, TactiCath SE, or TactiFlex SE, Abbott, Abbott Park, IL) at a power titrated to a maximum of 50 W.

In non‐DEMAP cases, VT ablation was performed at the critical isthmus site as determined by activation and entrainment mapping or identification of substrate based on the presence of LPs, LAVAs, DZs during sinus rhythm or RV pacing, or paced morphology similar to the VT with a long stimulus‐QRS interval. After ablation, remapping was performed to confirm elimination of abnormal signals and loss of capture at the ablated areas. In the non‐DEMAP group, ablation was performed at abnormal substrate and slow conduction areas, with the procedural endpoint of elimination to loss of capture. When feasible, ablation was further guided by activation mapping or pace mapping to confirm proximity to VT circuits.

In DEMAP cases, all areas identified as slow conduction zones within the scar and the scar border were ablated, and remapping was performed after ablation to confirm elimination of focused DEMAP slow conduction zones. If slow conduction persisted, additional ablation was performed until slow conduction zones were eliminated.

Procedural endpoints included VT non‐inducibility in the non‐DEMAP group. In the focused DEMAP group, the endpoints were VT non‐inducibility and elimination of the slow corridors identified through the focused DEMAP. VT non‐inducibility at the end of the procedure was assessed through programmed stimulation with up to three extrastimuli until refractoriness and/or a cycle length of 200 ms from the RV apex.

### 2.6 Clinical Follow‐Up

Patients were routinely followed at 3, 6, and 12 months postablation with clinical assessments, ICD interrogation, and remote monitoring. VT recurrences, cardiovascular hospitalizations, and mortality data were recorded. VT recurrence was defined as sustained monomorphic VT lasting > 30 s or appropriate ICD therapy, including anti‐tachycardia pacing or shocks. Cardiovascular hospitalizations included admission for VT, myocardial infarction, stroke, or heart failure. Time to first cardiovascular‐related hospitalization and AICD shock were assessed from the ablation date. Follow‐up time was measured from the date of ablation until an outcome was recorded or April 30, 2025, whichever came first. Patients lost to follow‐up were censored, and mortality was confirmed through electronic medical records or family communication.

### 2.7 Statistical Analysis

Baseline characteristics were summarized as mean ± standard deviation (SD) for continuous variables, and as frequencies and percentages for categorical variables. Differences between groups were assessed using the Student's *t*‐test for normally distributed continuous variables and the Wilcoxon rank‐sum test for non‐normally distributed variables. Categorical variables were compared using the chi‐square test or Fisher's exact test, as appropriate. Standardized mean differences (SMDs) were calculated to quantify balance between groups, with a value < 0.1 considered indicative of adequate balance. Covariate balance before and after weighting was assessed using SMDs.

To adjust for potential confounders, inverse probability of treatment weighting (IPTW) with stabilized weight was applied. Propensity scores were estimated using logistic regression with covariates including age, BMI, left ventricular ejection fraction (LVEF), antiarrhythmic drug use, cardiomyopathy type, history of previous ablation, left ventricular access methods, and mapping chamber. Stabilized IPTW were calculated to reduce variability and improve efficiency, ensuring covariate balance between treatment groups. Covariate balance was assessed after IPTW, and for any variables with residual imbalance (SMD ≥ 0.1), double adjustment was performed by including those variables in the outcome models.

Time‐to‐event outcomes were analyzed using stabilized‐IPTW Kaplan–Meier survival curves and cumulative incidence curves. The log‐rank test was used to compare survival distributions between groups, accounting for stabilized weights. For paired binary outcomes, McNemar's test was applied. All statistical analyses were conducted using Stata version 19 (StataCorp, College Station, TX). A two‐sided *p*‐value < 0.05 was considered statistically significant.

## 3 Results

### 3.1 Baseline Characteristics

A total of 57 patients underwent catheter ablation for scar‐related VT during the study period were included (25 patients [43.9%] assigned to the focused DEMAP group and 32 patients [56.1%] to the non‐DEMAP group). Baseline characteristics before IPTW are presented in Table 1. Between‐group balance was evaluated using standardized differences (STDs), with values less than 0.1 considered to indicate good balance.

**Table 1:** Baseline characteristics of the study population before inverse probability of treatment weighting (IPTW).

Baseline	Total (*n* = 57)	Non‐DEMAP (*n* = 32)	Focused DEMAP (*n* = 25)	STD
Age (mean ± SD)	64.30 ± 15.00	63.78 ± 16.10	64.96 ± 13.72	0.07
Race (*n*, %)				0.02
White	48 (84.21%)	27 (84.38%)	21 (84.00%)	
American Indian	2 (3.51%)	1 (3.12%)	1 (4.00%)	
Hispanic/Latin	1 (1.75%)	1 (3.12%)	0 (0.00%)	
Unknown	6 (10.53%)	3 (9.38%)	3 (12.00%)	
Sex (*n*, %)				0.10
Male	49 (85.96%)	27 (84.38%)	22 (88.00%)	
Female	8 (14.04%)	5 (15.62%)	3 (12.00%)	
BMI (mean ± SD)	29.69 ± 5.81	29.36 ± 6.17	30.11 ± 5.40	0.12
LVEF (mean ± SD)	37.89 ± 12.64	37.78 ± 12.41	38.04 ± 13.18	0.02
Devices (*n*, %)				0.04
Single AICD	8 (14.04%)	5 (15.62%)	3 (12.00%)	
Dual AICD	20 (35.09%)	10 (31.25%)	10 (40.00%)	
CRT‐D	19 (33.33%)	11 (34.38%)	8 (32.00%)	
No	10 (17.54%)	6 (18.75%)	4 (16.00%)	
Antiarrhythmic (*n*, %)				0.23
1 medication	29 (50.88%)	13 (40.62%)	16 (64.00%)	
2 medications	15 (26.32%)	11 (34.38%)	4 (16.00%)	
3 medications	2 (3.51%)	2 (6.25%)	0 (0.00%)	
No medication	11 (19.0%)	6 (18.75%)	5 (20.00%)	
Cardiomyopathy (*n*, %)				0.26
Ischemic	37 (64.91%)	19 (59.38%)	18 (72%)	
Nonischemic	20 (35.09%)	13 (40.62%)	7 (28%)	
Pre‐ablation (*n*, %)				0.07
No	47 (82.46%)	26 (81.25%)	21 (84.00%)	
Yes	10 (17.54%)	6 (18.75%)	4 (16.00%)	
LV access (*n*, %)				0.24
Transseptal	51 (89.47%)	28 (87.50%)	23 (92.00%)	
Retrograde	2 (3.51%)	1 (3.12%)	1 (4.00%)	
Both	2 (3.51%)	1 (3.12%)	1 (4.00%)	
No	2 (3.51%)	2 (6.25%)	0 (0.00%)	
Mapping chamber (*n*, %)				0.04
LV	40 (70.18%)	22 (68.75%)	18 (72.00%)	
RV	3 (5.26%)	2 (6.25%)	1 (4.00%)	
LV + RV	14 (24.56%)	8 (25.00%)	6 (24.00%)	

Abbreviations: AICD, automated implantable cardioverter defibrillator; BMI, body mass index; CRT‐D, cardiac resynchronization therapy with defibrillator; focused DEMAP, double extrastimuli mapping and ablation; ILAM, isochronal late activation mapping; LV, left ventricle; LVEF, left ventricular ejection fraction; LVZ, low‐voltage zone; PF, peak frequency; RV, right ventricle; SR, sinus rhythm; STD, standardized difference; VT, ventricular tachycardia.

The mean age was 64.96 ± 13.72 years in the focused DEMAP group and 63.78 ± 16.10 years in the non‐DEMAP group (STD = 0.07). The majority of patients in both groups were white (84.0% vs. 84.38%), with similar sex distribution (88.0% male in focused DEMAP vs. 84.38% in non‐DEMAP; STD = 0.10). Mean BMI and LVEF were also comparable (BMI: 30.11 ± 5.40 vs. 29.36 ± 6.17, STD = 0.12; LVEF: 38.04 ± 13.18% vs. 37.78 ± 12.41%, STD = 0.02).

Device type distribution showed a slightly higher proportion of dual AICD implants in the focused DEMAP group (40.0%) compared to the non‐DEMAP group (31.25%), with a STD of 0.04. Use of antiarrhythmic medications was generally higher in the focused DEMAP group, particularly single‐drug therapy (64.0% vs. 40.62%), and no patients in focused DEMAP received three medications (0% vs. 6.25% in non‐DEMAP), resulting in an overall STD of 0.23 for this category. At baseline, amiodarone was prescribed in 19 of 32 patients (59.4%) in the non‐DEMAP group and 10 of 25 patients (40.0%) in the DEMAP group, with no significant difference between groups (*p* = 0.18).

The underlying cardiomyopathy was ischemic in 72.0% of the focused DEMAP group and 59.38% of the non‐DEMAP group (STD = 0.26). Pre‐ablation procedures had similar frequencies (16.0% in focused DEMAP vs. 18.75% in non‐DEMAP; STD = 0.07). Most patients underwent transseptal access to the LV (92.0% in focused DEMAP vs. 87.5% in non‐DEMAP; STD = 0.24). Regarding mapping strategy, the LV was the predominant chamber in both groups (72.0% vs. 68.75%), with similar rates of combined LV and RV mapping (24.0% vs. 25.0%), and a STD of 0.04 for the mapping chamber. A summary of baseline characteristics after IPTW adjustment is provided in Table 2.

**Table 2:** Baseline characteristics of the study population after inverse probability of treatment weighting (IPTW).

Baseline	Total (*n* = 57)	Non‐DEMAP (*n* = 32)	Focused DEMAP (*n* = 25)	STD
Age (mean ± SD)	64.48 ± 14.50	64.71 ± 15.55	64.18 ± 13.31	0.03
Race (*n*, %)				0.03
White	48 (83.14%)	27 (82.87%)	21 (83.49%)	
American Indian	2 (3.63%)	1 (2.86%)	1 (4.63%)	
Hispanic/Latin	1 (1.44%)	1 (2.52%)	0 (0.00%)	
Unknown	6 (11.80%)	3 (11.74%)	3 (11.88%)	
Sex (*n*, %)				0.01
Male	49 (85.62%)	28 (85.70%)	21 (85.52%)	
Female	8 (14.38%)	4 (14.30%)	4 (14.48%)	
BMI (mean ± SD)	29.82 ± 5.79	29.78 ± 6.30	29.88 ± 5.18	0.02
LVEF (mean ± SD)	37.72 ± 12.78	36.87 ± 12.39	38.85 ± 13.46	0.01
Devices (*n*, %)				0.08
Single AICD	7 (13.48%)	4 (14.09%)	3 (12.67%)	
Dual AICD	20 (34.81%)	10 (30.22%)	10 (40.90%)	
CRT‐D	20 (34.07%)	12 (37.86%)	8 (29.05%)	
No	10 (17.63%)	6 (17.82%)	4 (17.38%)	
Antiarrhythmic (*n*, %)				0.08
1 medication	29 (51.60%)	14 (42.98%)	15 (63.03%)	
2 medications	16 (28.08%)	12 (37.74%)	4 (15.28%)	
3 medications	2 (3.26%)	2 (5.72%)	0 (0.00%)	
No medication	10 (17.06%)	4 (13.56%)	6 (21.69%)	
Cardiomyopathy (*n*, %)				0.03
Ischemic	38 (66.43%)	21 (65.73%)	17 (67.35%)	
Nonischemic	19 (33.57%)	11 (34.27%)	8 (32.65%)	
Pre‐ablation (*n*, %)				0.07
No	47 (83.07%)	26 (81.83%)	21 (84.72%)	
Yes	10 (16.93%)	6 (18.17%)	4 (15.28%)	
LV access (*n*, %)				0.10
Transseptal	51 (89.47%)	29 (93.75%)	22 (88.00%)	
Retrograde	3 (5.26%)	1 (2.08%)	2 (8.00%)	
Both	2 (3.51%)	1 (2.08%)	1 (4.00%)	
No	1 (1.75%)	1 (2.08%)	0 (0.00%)	
Mapping chamber (*n*, %)				0.08
LV	41 (72.93%)	23 (71.55%)	18 (74.75%)	
RV	3 (4.38%)	1 (4.01%)	2 (4.87%)	
LV + RV	13 (22.69%)	8 (24.44%)	5 (20.38%)	

Abbreviations: AICD, automated implantable cardioverter defibrillator; BMI, body mass index; CRT‐D, cardiac resynchronization therapy with defibrillator; focused DEMAP, focused double extrastimuli mapping and ablation; ILAM, isochronal late activation mapping; LV, left ventricle; LVEF, left ventricular ejection fraction; LVZ, low‐voltage zone; PF, peak frequency; RV, right ventricle; SR, sinus rhythm; STD, standardized difference; VT, ventricular tachycardia.

### 3.2 Electrophysiological Study Data

The mean VT cycle length was 353.03 ± 90.73 ms. Extrastimuli cycle lengths were 562.00 ± 52.59 ms for S1, 299.20 ± 31.87 ms for S2, and 267.40 ± 33.76 ms for S3. Baseline programmed stimulation was performed in all patients. Inducible VT was observed in 28 of 32 patients (87.5%) in the non‐DEMAP group, with 46 VTs induced, and in 20 of 25 patients (80.0%) in the DEMAP group, with 43 VTs induced. Hemodynamically tolerated activation mapping was feasible in 17 of 46 VTs (37%) in the non‐DEMAP group and 15 of 43 VTs (35%) in the DEMAP group. During sinus rhythm, DZs were distributed across various myocardial substrates, occurring in normal myocardium in 51.70% of cases, in the border zone in 44.80%, and within scar in only 3.50%. In contrast, focused DEMAP mapping revealed a different pattern, with DZs predominantly located in the border zone (75.80%), followed by normal tissue (20.60%) and scar (3.50%). Regarding spatial correlation with the VT isthmus, 26 of 70 total DZs identified during sinus rhythm ILAM (37.1%) were concordant with the VT isthmus. In comparison, 22 of 29 DZs identified using focused DEMAP (75.9%) matched the VT isthmus with a statistically significant difference (*p* = 0.01). These findings are summarized in Table 3.

**Table 3:** Mapping characteristics in focused DEMAP group.

	Sinus rhythm mapping	Focused DEMAP
Width of ILAM window (ms)	350.80 ± 64.22	294.08 ± 23.19
Total points collected		
Unipolar	12 812 ± 5594	377 ± 225
Bipolar	18 885 ± 8266	570 ± 339
Omnipolar	28 477 ± 12 470	852 ± 512
DZ burden (%)		
Normal tissue voltage	51.7	20.6
Border zone	44.8	75.8
Scar	3.5	3.5
VT isthmuses/number of DZs (%)	37.1	75.9

*Note:* Values are number (%), mean (± 1 standard deviation).

Abbreviations: DEMAP, double extrastimulus mapping; DZ, deceleration zone; ILAM, isochronal late activation mapping; VT, ventricular tachycardia.

VT isthmus locations were predominantly within the scar border zone across all mapping modalities. With unipolar mapping, 88.00% of isthmuses were localized to the border zone and 12.00% to scar, with no sites in normal tissue. Bipolar mapping showed 74.10% in the border zone, 18.50% in the scar, and 7.40% in normal myocardium. Omnipolar mapping (OT) identified isthmus regions in the border zone in 77.80% of cases, with 7.40% in scar and 14.80% in normal tissue. In the DEMAP group (*n* = 25), critical isthmuses were defined by concealed entrainment with good PPI in 15 patients (60%), by termination during ablation in 8 patients (32%), and by good pace match with long stim‐QRS in 2 patients (8%). In the non‐DEMAP group (*n* = 32), critical isthmuses were defined by concealed entrainment with good PPI in 17 patients (53%), by termination during ablation in 6 patients (19%), and by good pace match in 9 patients (28%).

**Figure 3:**
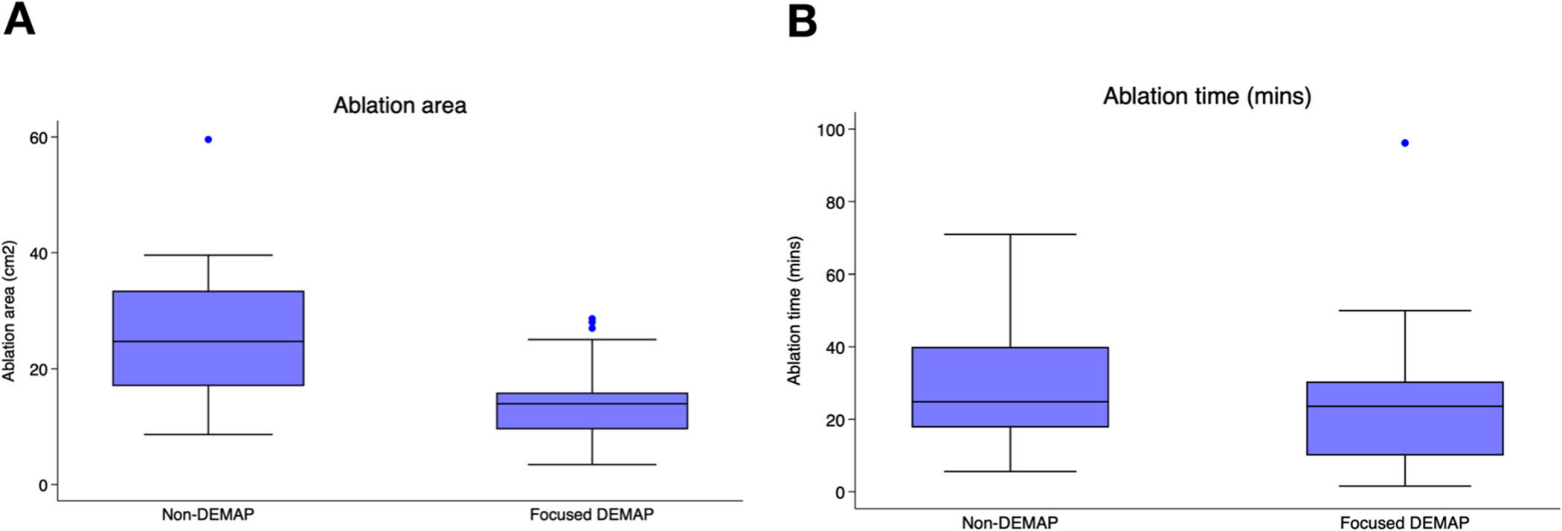
Procedural metrics comparing non‐DEMAP and focused DEMAP groups. (A) Boxplot illustrating the ablation area (cm^2^) between groups. Focused DEMAP was associated with a significantly smaller ablation area (14.19 ± 7.23 cm^2^) compared to non‐DEMAP (26.03 ± 11.86 cm^2^) (*p* < 0.001), suggesting improved lesion efficiency. (B) Boxplot showing ablation time in minutes. Although ablation duration tended to be shorter in the focused DEMAP group (24.91 ± 19.38 min) compared to non‐DEMAP (30.94 ± 17.62 min), the difference was not statistically significant (*p* = 0.26).

At the end of the procedure, VT termination by non‐inducibility was achieved in 22 of 32 patients (68.75%) in the non‐DEMAP group and 20 of 25 patients (80.00%) in the focused DEMAP group, with no statistically significant difference between groups (*p* = 0.38). The average procedure time, measured from sheath insertion to sheath removal, was 193.09 ± 43.92 min in the non‐DEMAP group and 189.72 ± 43.12 min in the DEMAP group (*p* = 0.77), indicating no significant difference in overall procedure duration between the two approaches (Figure 3A). In the DEMAP group, DZs represented ~4.6% of the DEMAP‐mapped area per case, and all identified zones were ablated. Four of 25 patients (16%) remained inducible after elimination of DEMAP‐identified zones; two underwent additional ablation at conventional substrate sites (AMC and kissing lesion at the LV septum), while two were cardioverted without further ablation. In the non‐DEMAP group, 9 of 32 patients (28%) remained inducible, and all underwent additional ablation guided by conventional substrate mapping.

There was no significant difference in the rate of VT termination between groups (non‐inducibility: 80.00% in the DEMAP group vs. 68.75% in the non‐DEMAP group; *p* = 0.38). Cardioversion was required in 20.00% of patients in the DEMAP group and 31.25% in the non‐DEMAP group. Notably, ablation duration tended to be shorter in the DEMAP group (24.91 ± 19.38 min) compared to the non‐DEMAP group (30.94 ± 17.62 min), though this did not reach statistical significance (*p* = 0.26). However, the total area ablated was significantly smaller in the DEMAP group (14.19 ± 7.23 cm²) than in the non‐DEMAP group (26.03 ± 11.86 cm², *p* < 0.001), suggesting that the focused DEMAP strategy may allow for more efficient lesion targeting with less overall tissue ablation (Figure 3B).

### 3.3 Acute and Long‐Term Procedural Outcomes

Procedural complications occurred in 2 of 32 patients (6.2%) in the non‐DEMAP group and none in the DEMAP group. In the non‐DEMAP group, complications included pericardial effusion requiring pericardiocentesis, complete atrioventricular block due to an abnormal substrate close to the conduction system requiring CRT‐D upgrade. No procedure‐related deaths or cerebrovascular events occurred. No major complications were observed in the DEMAP group.

**Figure 4:**
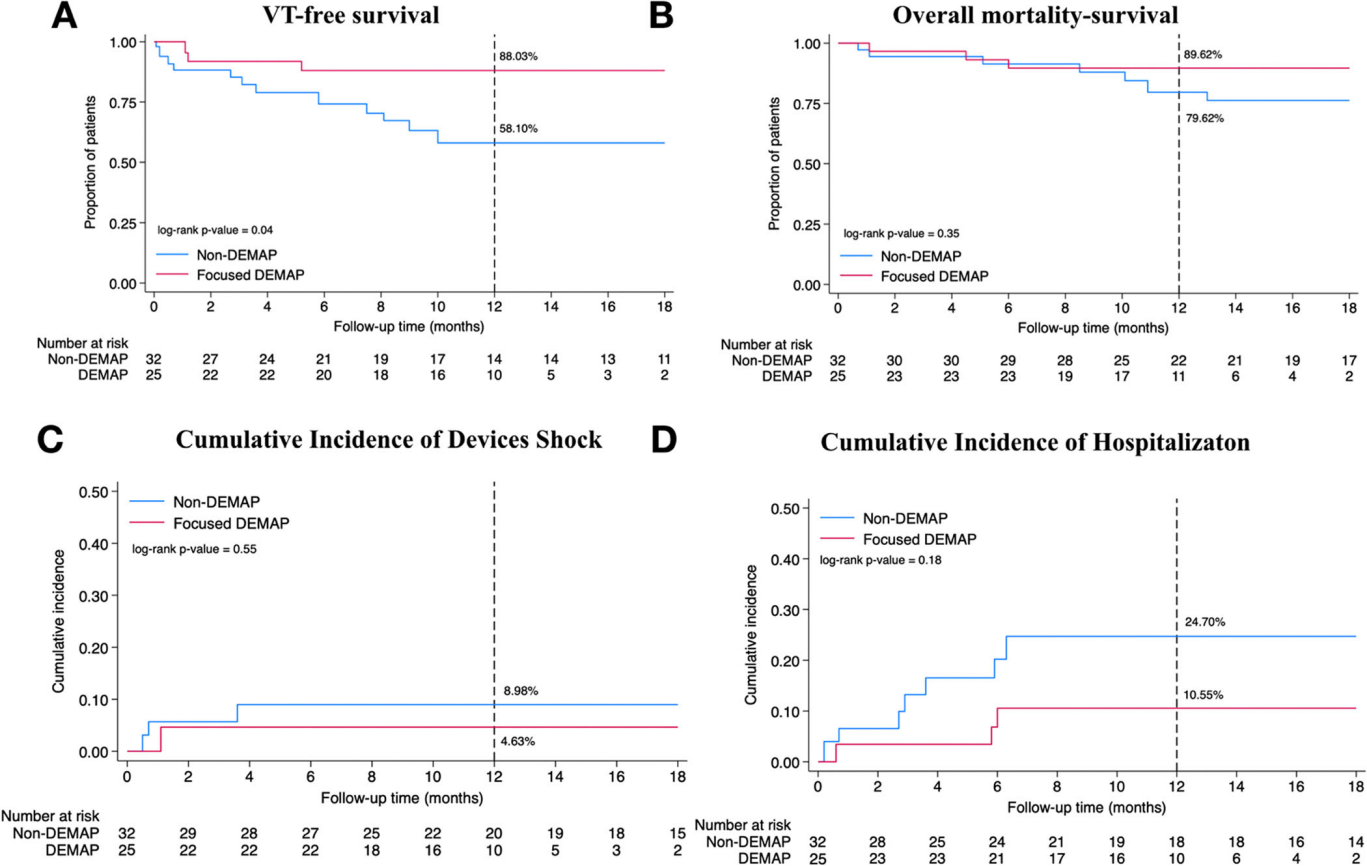
Kaplan–Meier curves and cumulative incidence plots for clinical outcomes. This figure presents Kaplan–Meier survival curves and cumulative incidence plots comparing focused DEMAP and non‐DEMAP groups over a 12‐month follow‐up period. (A) Kaplan–Meier curve for VT‐free survival, demonstrating significantly higher VT‐free survival in the focused DEMAP group (88.03%) compared to the non‐DEMAP group (58.10%) (log‐rank *p* = 0.04). (B) Kaplan–Meier curve for overall survival, showing a trend toward lower mortality in the focused DEMAP group (89.62%) versus the non‐DEMAP group (79.62%) (log‐rank *p* = 0.35). (C) Cumulative incidence plot for AICD shock therapy, indicating a lower incidence in the focused DEMAP group (4.60%) compared to the non‐DEMAP group (8.98%), though not statistically significant (log‐rank *p* = 0.55). (D) Cumulative incidence plot for cardiovascular‐related hospitalization, revealing a lower hospitalization rate in the focused DEMAP group (10.55%) versus the non‐DEMAP group (24.70%) (log‐rank *p* = 0.18).

At 12‐month follow‐up, VT‐free survival was significantly higher in the focused DEMAP group compared to the non‐DEMAP group. Specifically, 88.03% of patients in the focused DEMAP group remained free from VT recurrence, whereas only 58.10% of patients in the non‐DEMAP group achieved VT‐free survival. This difference reached statistical significance, with a log‐rank p‐value of 0.04, as illustrated in Figure 4A. The survival curves showed early and sustained divergence between groups, suggesting a possible benefit of the focused double extrastimuli mapping approach.

All‐cause mortality at 12 months was 10.38% in the focused DEMAP group and 20.38% in the non‐DEMAP group, corresponding to survival rates of 89.62% and 79.62%, respectively. Although the absolute difference was notable, this outcome did not reach statistical significance (log‐rank *p* = 0.35) (Figure 4B). Regarding hospitalization due to cardiovascular causes, the focused DEMAP group experienced a lower event rate. Hospitalization occurred in 10.55% of patients who underwent DEMAP‐guided ablation, compared to 24.70% in the non‐DEMAP group. While this trend favored the focused DEMAP approach, the difference was not statistically significant (log‐rank *p* = 0.18) (Figure 4D). Incidence of AICD shock within the 12‐month period was also numerically lower in the DEMAP group. Only 4.63% of patients in the focused DEMAP group experienced a device shock compared to 8.98% in the non‐DEMAP group. However, this difference did not meet statistical significance (log‐rank *p* = 0.55) (Figure 4C).

**Figure 5:**
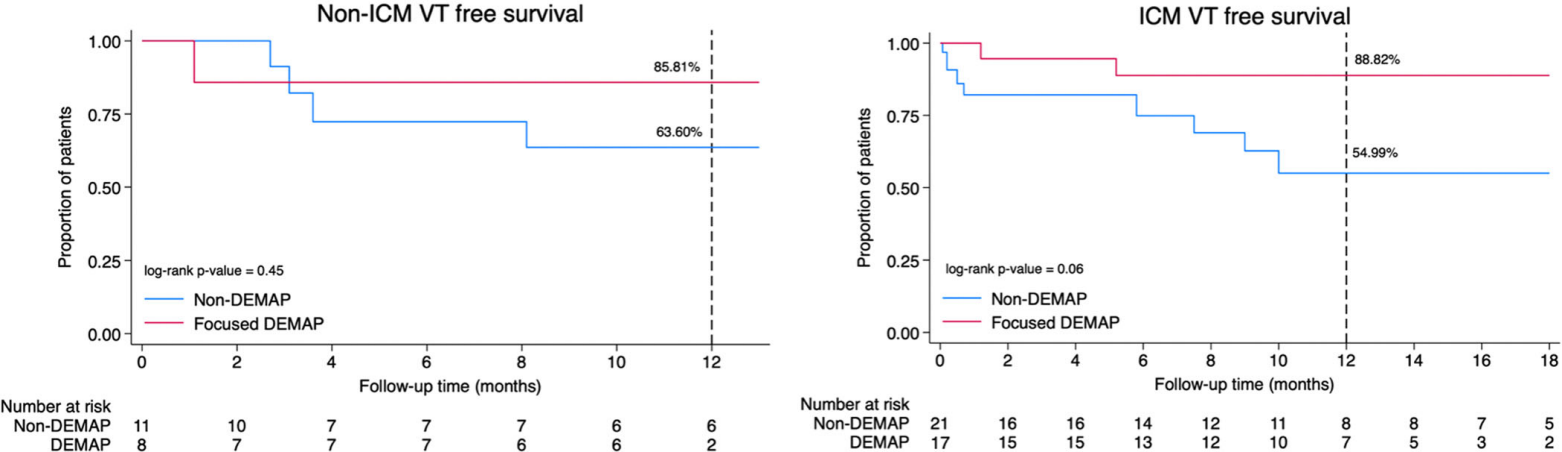
Subgroup analysis of VT‐free survival in ICM and NICM. Kaplan–Meier curves comparing 12‐month VT‐free survival between focused DEMAP and non‐DEMAP groups, stratified by cardiomyopathy type. (left) (NICM) VT‐free survival was higher in the focused DEMAP group (85.81%) versus the non‐DEMAP group (63.60%), although the difference was not statistically significant (log‐rank *p* = 0.45). (right) (ICM) Focused DEMAP showed a numerically higher VT‐free survival rate (88.82%) compared to non‐DEMAP (54.99%), with a trend toward significance (log‐rank *p* = 0.06).

We performed a subgroup analysis stratified by cardiomyopathy type to assess the impact of the focused DEMAP in ICM versus NICM. In the ICM subgroup, patients who underwent focused DEMAP‐guided ablation demonstrated a numerically higher VT‐free survival rate at 12 months compared to the non‐DEMAP group (88.82% vs. 54.99%, log‐rank *p* = 0.06). Similarly, in the NICM subgroup, the focused DEMAP group also exhibited a higher VT‐free survival rate (85.81% vs. 63.60%), although this difference did not reach statistical significance (log‐rank *p* = 0.45). These findings are illustrated in Figure 5.

## 4 Discussion

### 4.1 Main Findings

This study demonstrates that the focused DEMAP approach to map and ablate VT may improve VT substrate identification and post‐procedural outcomes. This approach does not appear to prolong study duration due to the limited and focused mapping performed within areas of scar. By selectively applying extrastimuli in regions of interest, the focused DEMAP enhances the correlation between DZs and VT circuits and the identification of arrhythmogenic substrates. Importantly, this method may also improve the specificity of ablation targets by eliminating nonconducting regions during extrastimulus mapping. Focused DEMAP was associated with improved VT‐free survival and lower post‐procedural hospitalization rates, suggesting better long‐term arrhythmia control compared to conventional mapping approaches. These outcomes were achieved with a smaller area of ablated tissue in the non‐DEMAP group, likely due to enhanced identification of critical parts of VT circuits with this method. To our knowledge, this is the first study to apply omnipolar electrograms for functional VT substrate mapping using a double extrastimuli protocol, highlighting both the technical feasibility and clinical relevance of this approach.

### 4.2 The Role of Extrastimulus Mapping in VT Ablation

Extrastimulus‐based functional substrate mapping has been introduced as an alternative strategy to unmask slow conduction channels that remain undetectable with traditional substrate mapping, as illustrated in Figure 2. Prior studies have demonstrated that extrastimuli enhance substrate identification by revealing functionally relevant conduction channels that are otherwise concealed with conventional mapping techniques. By shortening the diastolic interval, modifying local refractoriness, and leveraging decremental conduction properties within abnormal myocardial tissue, extrastimuli expose hidden slow‐conduction pathways, exaggerate conduction delays, and induce functional block within scarred myocardium [9, 10]. This approach improves sensitivity in identifying the VT isthmus while maintaining specificity, outperforming standard sinus rhythm mapping and single extrastimulus pacing [11, 12]. The systematic application of double extrastimuli across the entire chamber has demonstrated substantial benefits in refining scar characterization and localizing arrhythmogenic zones [6]. A recent systematic review and meta‐analysis by Wilnes et al. [13]. provides the current evidence in favor of extrastimuli‐assisted functional mapping in VT ablation. Their findings demonstrate that dynamic mapping strategies, particularly those involving extrastimuli (e.g., S3 protocols), are associated with lower VT recurrence rates. While two studies in the analysis (Acosta et al. [14] and Guichard et al. [15]) used global S3 extrastimuli application, our study introduces a focused approach that targets specific regions. This external validation supports the physiologic relevance of stimulation‐induced substrate unmasking and underscores the clinical value of incorporating extrastimuli in substrate‐based VT ablation. Global extrastimuli mapping may increase procedural time and expose patients to repetitive pacing, which may lead to potential proarrhythmia. Considering its value and the fact that localized substrate often causes VT, a more focused approach could offer similar benefits while minimizing time and associated risks.

In this study, we utilized focused DEMAP in regions of interest, allowing us to unmask slow conduction areas with greater specificity for critical isthmuses. This strategy allowed more targeted ablation, and abolition of the slow conduction zones with S3 mapping was used as an endpoint at the end of the procedure in addition to the non‐inducibility endpoint.

### 4.3 The Impact of the Focused DEMAP on Substrate Mapping and Clinical Success

The focused DEMAP employs a selective double extrastimuli approach targeting DZs, border zones, and low‐voltage regions—areas most likely to contain critical conduction pathways. This strategy facilitates more precise and efficient functional substrate mapping [16, 17]. Unlike sinus rhythm‐based mapping, which may fail to reveal functionally significant conduction channels, this method delineates key regions essential for VT maintenance while minimizing unnecessary ablation. It is particularly advantageous in patients for whom VT activation mapping is unfeasible due to hemodynamic instability, allowing for a more targeted ablation strategy without significantly prolonging procedural time.

Voltage mapping remains central to VT substrate identification, with bipolar electrograms traditionally used to define scar and border zones. However, bipolar voltage measurements are highly dependent on the wavefront orientation relative to the dipole axis, which can introduce variability in voltage readings and lead to misclassification of scar regions [18]. OT offers an alternative by providing orientation‐independent voltage measurements, improving the accuracy of substrate delineation [19, 20]. Studies have demonstrated that OT maps yield higher voltage values in areas classified as scar by bipolar mapping, refining the distinction between scar and border zones [21]. Integrating OT into the focused DEMAP may further enhance substrate identification and improve ablation efficacy by avoiding unnecessary lesion delivery in noncritical areas.

Previous studies have reported varying VT‐free survival rates depending on the mapping and ablation strategies employed. Jais et al. demonstrated that a LAVA‐targeted ablation approach resulted in approximately 60% VT‐free survival at 1 year following complete elimination of LAVAs [2]. Similarly, Ascione et al. utilized OT to ablate LP regions, achieving a comparable VT‐free survival rate of 60% at 1 year [21]. Aziz et al. introduced a DZ‐based ablation strategy, reporting an overall VT‐free survival rate of 70%, with outcomes stratified by 80% in ICM and 63% in NICM [3]. More recently, Guichard et al. demonstrated an 80% VT‐free survival rate using the S3 protocol with whole‐chamber mapping [6].

These findings reinforce the notion that systematic, functional mapping approaches enhance VT substrate identification and improve long‐term procedural outcomes. The evolution from focal ablation strategies, such as LAVA and LP elimination, to broader mapping techniques that integrate both anatomic and functional assessments highlights the advantages of a comprehensive approach to VT ablation. Our findings support that a focused S3 extrastimulus mapping increases the specificity of DZs as critical VT isthmuses, and an ablation approach guided by the S3 mapping improves clinical outcomes after VT ablations compared to traditional mapping strategies. This procedural efficacy was achieved without significantly increasing procedure time and, more importantly, with less cardiac tissue ablated. Although mortality and hospitalization differences did not reach statistical significance, the trend favors improved long‐term control. Larger, prospective studies across varied mapping systems will be necessary to validate and extend these findings.

### 4.4 Study Limitations

This study highlights the potential of the focused DEMAP in improving substrate identification for VT ablation. However, several limitations should be considered when interpreting the findings. One major limitation is the sample size, which may have impacted the statistical power of some comparisons. While the study demonstrated a trend toward improved outcomes in the focused DEMAP group, including a lower rate of VT recurrence and reduced hospitalization, some differences did not reach statistical significance. Another consideration is patient selection bias for the procedure and for the procedural approach. The study population consisted of consecutive patients undergoing VT ablation at a single institution, which may limit generalizability. This consecutive enrollment provides real‐world understanding but resulted differences between the comparison groups. For example, there were differences in cardiomyopathy type (ischemic vs. nonischemic), underlying scar patterns, and VT morphology that could influence how effectively the focused DEMAP identifies arrhythmogenic substrate. Future research should explore the protocol's effectiveness across a more diverse patient cohort.

Finally, technology‐specific considerations should be addressed. The focused DEMAP was performed using EnSite X with OT, which offers high‐resolution voltage and electrogram annotation capabilities. However, it remains unclear whether the protocol would yield similar results using other mapping systems. Future validation across different electroanatomic platforms will be essential for broader clinical adoption.

## 5 Conclusion

This study demonstrates that the focused DEMAP enhances VT substrate identification by selectively applying double extrastimuli in regions of interest, including DZs, border zones, and low‐voltage areas. By improving the correlation between DZs and VT isthmuses, the protocol refines substrate‐based ablation strategies without the need for extensive scar homogenization. The findings suggest that the focused DEMAP may offer several procedural and long‐term clinical advantages compared to conventional mapping techniques.

Further validation across different mapping systems and larger patient populations will be essential to fully define its role in modern VT ablation strategies.

## Conflicts of Interest

The authors declare no conflicts of interest.
